# Solutions for Africa's Health Workforce Crisis through Country Based Research

**DOI:** 10.1186/1478-4491-12-S1-I1

**Published:** 2014-05-12

**Authors:** Francis Omaswa

**Affiliations:** 1 Chair, African Platform on Human Resources for Health; Founding Executive Director, Global Health Workforce Alliance, Kampala, Uganda

## 

Africa is currently undergoing a transition from being a continent in deep trouble to a continent with a new hope characterized by more democracy, public accountability, and steady economic growth within a global environment that supports equity and social justice for all.

In the pursuit of agreed global and national health goals, the role of the health workforce is absolutely critical. Health care provision is a service industry that depends very much on the people who provide the services to clients. Yet over the years, especially during the difficult times in Africa, attention and support to the health workforce was not accorded the priority that it deserves. Emphasis was instead accorded to the provision of commodities, procurement of equipment, and construction of facilities. Health workforce was considered to be too complicated and not sustainable for the international community to engage and was primarily left as the responsibility of national and local governments.

This period of deliberate and regrettable neglect left Africa at the bottom of the global league table, experiencing a disproportionate global disease burden of 24% and the least share of health workforce at only 3% (World Health report 2006) [1]. The same study showed that out of 57 countries globally classified as having critical health workforce numbers, 36 of them were in sub-Saharan Africa. Another study, the Sub-Saharan African Medical School Survey showed that sub-Saharan Africa, with a total population 800 million, was training only 6 000 medical doctors, a number that is similar to those trained by OECD countries with populations of only 60 million [2].

Fortunately, the situation has changed for the better, fueled by a number of global studies; pressure from political and professional leaders from African and other low income countries that are suffering brain drain as well as the activism to increase access to treatment for HIV and AIDS. Today, there is heightened global interest in health workforce issues, the Global Health Workforce Alliance was established at the World Health Organization to coordinate the response to what is now recognized as a global crisis that is characterized by widespread shortages, mal-distribution, and poor working conditions. Three global forums have been held on the topic and the first, in 2008, adopted the Kampala Declaration and Agenda for Global Action that guides the global and national responses.

It is against this background that the studies featured in this supplement, carried out in Africa through partnerships between Africans and their international associates, is truly welcome. The reports are being published at an opportune time when Africa and the international community are in need of essential information and evidence to make the right decisions on the national, regional, and global response. These studies will be able to guide policy makers and implementers on the ground on resource allocation and local problem solving. I congratulate the research teams funded through the Africa Health Systems Initiative - Support to African Research Partnerships (AHSI-RES) program for this outstanding work and I am pleased and honored to have been invited to advise on some of the studies in this report and to contribute this foreword.

## Competing interests

The author declares that he has no competing interests.

## Declarations

This article has been published as part of *Human Resources for Health* Volume 12 Supplement 1, 2014: Addressing the human resources for health crisis through task-shifting and retention: results from the Africa Health Systems Initiative's research component. The full contents of the supplement are available online at http://www.human-resources-health.com/supplements/12/S1
